# Influenza Vaccination Generates Cytokine-Induced Memory-like NK Cells: Impact of Human Cytomegalovirus Infection

**DOI:** 10.4049/jimmunol.1502049

**Published:** 2016-05-27

**Authors:** Martin R. Goodier, Ana Rodriguez-Galan, Chiara Lusa, Carolyn M. Nielsen, Alansana Darboe, Ana L. Moldoveanu, Matthew J. White, Ron Behrens, Eleanor M. Riley

**Affiliations:** * Department of Immunology and Infection, London School of Hygiene and Tropical Medicine, London WC1E 7HT, United Kingdom;; † MRC International Nutrition Group, Medical Research Council, The Gambia Unit, London School of Hygiene and Tropical Medicine, London WC1E 7HT, United Kingdom; and; ‡ Department of Clinical Research, London School of Hygiene and Tropical Medicine, London WC1E 7HT, United Kingdom

## Abstract

Human NK cells are activated by cytokines, immune complexes, and signals transduced via activating ligands on other host cells. After vaccination, or during secondary infection, adaptive immune responses can enhance both cytokine-driven and Ab-dependent NK cell responses. However, induction of NK cells for enhanced function after in vitro exposure to innate inflammatory cytokines has also been reported and may synergize with adaptive signals to potentiate NK cell activity during infection or vaccination. To test this hypothesis, we examined the effect of seasonal influenza vaccination on NK cell function and phenotype in 52 previously unvaccinated individuals. Enhanced, IL-2–dependent, NK cell IFN-γ responses to Influenza A/California/7/2009 virus were detected up to 4 wk postvaccination and higher in human CMV (HCMV)-seronegative (HCMV^−^) individuals than in HCMV-seropositive (HCMV^+^) individuals. By comparison, robust NK cell degranulation responses were observed both before and after vaccination, due to high titers of naturally occurring anti-influenza Abs in human plasma, and did not differ between HCMV^+^ and HCMV^−^ subjects. In addition to these IL-2–dependent and Ab-dependent responses, NK cell responses to innate cytokines were also enhanced after influenza vaccination; this was associated with proliferation of CD57^−^ NK cells and was most evident in HCMV^+^ subjects. Similar enhancement of cytokine responsiveness was observed when NK cells were cocultured in vitro with Influenza A/California/7/2009 virus, and this was at least partially dependent upon IFN-αβR2. In summary, our data indicate that attenuated or live viral vaccines promote cytokine-induced memory-like NK cells and that this process is influenced by HCMV infection.

## Introduction

Lymphoid and myeloid cells can be primed or trained during pathogen exposure, leading to enhanced responses on reinfection (1, 2). The initial inflammatory cytokine response to infection shapes the subsequent immune response, different classes of pathogen inducing characteristic cytokine signatures, with lasting consequences for innate as well as adaptive cells (1). In the case of viral infections, different combinations and kinetics of innate cytokines, including IFN-α, IL-12, IL-15, and IL-18, activate NK cells within the first few hours and days of infection (3–6). These innate cytokines can also preactivate NK cells, leading to the formation of memory-like NK cells with enhanced capacity for cytokine production, degranulation, and target cell lysis (7–10). In mice, responses of cytokine preactivated NK cells are maintained after homeostatic proliferation and can persist for months after adoptive transfer, demonstrating further key features of immune memory formation (7, 11). These memory-like NK cells contrast with those that have received ongoing short duration stimulation with cytokines such as IL-15 (12–14). Human NK cells preactivated in vitro by cocktails of IL-12, IL-15, and IL-18 and rested for up to 21 d subsequently produce more IFN-γ after restimulation, and this phenotype is maintained after proliferative expansion (8). Whether cytokine-induced NK cell preactivation occurs in vivo during human infection or vaccination and, if so, how long this preactivated state persists and whether it potentiates the adaptive response is unknown. For example, influenza infection generates a characteristic systemic cytokine signature comprising IFN-α, IL-15, and IL-10 (3, 15–17), but it is not known whether this is sufficient to preactivate NK cells in vivo. Modest enhancement of IFN-γ (and IL-22) responses of mixed PBMC to heterologous bacterial pathogens has been reported up to 3 months after bacillus Calmette-Guérin vaccination and has been postulated as the mechanism underlying the long-term, nonspecific effects of bacillus Calmette-Guérin vaccines, but the sources of these cytokines are unknown (18, 19).

The molecular basis for generation of cytokine-induced memory-like NK is also, as yet, incompletely understood. Upregulation of CD25 (IL-2Rα) is characteristic of memory-like NK cells in humans (20) and in mice (21) and has also been reported shortly after influenza vaccination (22). CD25 upregulation on NK cells after vaccination and consequent enhanced sensitivity to IL-2 is associated with markedly increased, T cell/IL-2–dependent, NK cell IFN-γ and degranulation responses to vaccine Ags but would not easily explain enhanced responsiveness to other cytokines (23–26). Additionally, preactivation of NK cells with a mixture of IL-15 plus IL-12 plus IL-18 leads to epigenetic modifications including complete demethylation of the conserved upstream noncoding sequences of the IFN-γ gene (27). Interestingly, similar modifications are reported in the expanded NKG2C^hi^ NK cell subset in human CMV (HCMV)–infected subjects (27), suggesting that signaling through NKG2C (mediated, for example by HLA-E/CMV peptide complexes), with or without additional cytokine stimuli, also results in sustained functional modification of NK cells. The functional characteristics and longevity of cytokine-induced memory-like NK cells may therefore depend on an individual’s HCMV infection status. Moreover, the precise mixture of activating cytokines is important with IL-15 plus IL-12, IL-15 plus IL-18, or IL-15 alone leading to only partial demethylation of the *IFNG* locus (27).

In order to begin to understand the in vivo relevance of cytokine-induced memory-like NK cells in humans, we have characterized human NK cells before and after vaccination with either killed or live attenuated influenza viruses. We observe upregulation of NK cell responses to influenza virus after vaccination, with kinetics directly reflected in the ability of these cells to respond to innate cytokines. Moreover, the threshold for vaccine-induced effects is much higher in HCMV-seropositive (HCMV^+^) individuals, resulting in reduced responsiveness to vaccines. We also demonstrate, for the first time to our knowledge, that influenza virus can preactivate human NK cells in vitro, and this effect is at least partially dependent upon IFN-αβR2 mediated signals.

## Materials and Methods

### Subject recruitment and sample collection

Fifty-two healthy adult volunteers (median age 37.5 y; range 21–63 y) were enrolled into the study after providing informed consent. The study was approved by the ethical committee of the London School of Hygiene and Tropical Medicine (reference number 6237). None of the subjects had been previously vaccinated against influenza, and none had experienced influenza-like symptoms during the previous 6 mo. Subjects were randomly assigned to receive a single dose of 2012–2013 seasonal trivalent influenza vaccine (TIV) by the intradermal (ID; Intanza; Sanofi Pasteur MSD), i.m. (Split Virion BP; Sanofi Pasteur MSD), or intranasal (IN; Fluenz; AstraZeneca) routes. Randomization was structured so that the three arms of the study could be matched according to age and gender. The i.m. and ID vaccines contain chemically inactivated virus, whereas the IN vaccine contains live attenuated virus. The vaccines were all preservative free and not adjuvanted.

Heparinized blood was collected prior to (baseline sample) and 2, 4, and 12 wk after vaccination. Plasma samples were stored for assay of Abs to influenza and HCMV and for use in autologous cell cultures. PBMC were separated by standard Histopaque (Sigma-Aldrich) gradient centrifugation and processed within 4 h of blood collection for immediate (ex vivo) flow cytometry. Remaining cells were cryopreserved at 1 × 10^7^ cells/ml in RPMI 1640, 40% FCS, and 10% DMSO (Sigma-Aldrich), within 4 h of blood collection. Cells were stored for 18 h at −80°C in Nalgene cryoboxes with isopropanol coolant prior to transfer to liquid nitrogen for longer-term storage (25, 28).

### Cell culture conditions, NK cell activation, and IFN-α measurement

For each individual vaccinee, cells collected at baseline and at each postvaccination time point were tested side-by-side. Cryopreserved PBMC were thawed, washed, and counted on Fastread counting slides (Immune Systems), as previously described with median yield of 56% and viability by trypan blue exclusion of 98% (25, 26, 28). Cells were rested for 4–6 h, in the absence of exogenous cytokines, prior to stimulation. Briefly, 2 × 10^5^ PBMC were cultured for a total of 6 h (for T cell assays) or 18 h (to enable detection of T cell–dependent NK cell responses) in culture medium alone or with inactivated TIV (Split Virion BP; Sanofi Pasteur MSD) or inactivated Influenza A/California/7/2009 (H1N1) influenza virus (vNYMC-X179A; National Institute for Biological Standards and Control, Potters Bar, U.K.) in the presence or absence of low concentrations of cytokines (LCC), IL-12 (PeproTech, London, U.K.; 12.5 pg/ml) plus IL-18 (R&D Systems, Oxford, U.K.; 10 ng/ml). Cells incubated in culture medium alone or in LCC only were used as internal negative controls. Cells were also stimulated with MHC class I–deficient K562 cells (PBMC: K562 ratio of 2:1) or high concentrations of cytokines (HCC), IL-12 (5 ng/ml) plus IL-18 (50 ng/ml). GolgiStop (containing Monensin; 1/1500 concentration; BD Biosciences, Oxford, U.K.) and GolgiPlug (containing Brefeldin A; 1/1000 final concentration; BD Biosciences) were added after 3 h for T cell assays and after 15 h for NK cell assays. All assays were performed in 10% pooled AB serum, batch tested for performance in NK cell assays (Sigma-Aldrich) unless otherwise stated. In vitro neutralization experiments were performed using a rat anti-human IL-2 Ab (rat IgG2a; clone MQ1-17H12,NA/LE; BD Biosciences) or a rat IgG2a control reagent (eBioscience). To determine the role of influenza-specific IgG on NK cell responses, pooled AB plasma was depleted of IgG using a protein G Sepharose column (Millipore), as previously described (25). IFN-α was measured in tissue culture supernatants using a commercially available ELISA kit (PBL Assay Science).

### In vitro preactivation of NK cells with influenza virus

Preactivation of NK cells in vitro was performed essentially as previously described (8). PBMC (2 × 10^6^/ml) were cultured in 1 ng/ml IL-15 alone (control) or in IL-15 combined with either inactivated influenza H1N1 virus or HCC (IL-12 5 ng/m plus IL-18 50 ng/ml). After 16 h, cells were washed three times and maintained for a further 14 d in medium containing 1 ng/ml IL-15, replacing the IL-15–enriched medium every 2 to 3 d. Preactivated cells and control cells were restimulated for 6 or 18 h with different concentrations of IL-12 combined with either IL-18 or IL-15. To test for cytokine dependence of the preactivation process, neutralizing Abs to IL-2 (see *Cell culture conditions, NK cell activation, and IFN-α measurement*), IL-12 (clone NA/LE, C8.6, mouse IgG1 [mIgG1]; BD Biosciences), or IL-18 (clone 125-2H, mIgG1; R&D Systems) or blocking Ab against IFN-αβR2 (clone MMHAR2, mIgG2a; Merck Millipore) were added to H1N1-stimulated cultures. Isotype-matched control reagents were used as controls (BD Biosciences).

### Estimation of plasma anti-influenza IgG and anti-HCMV IgG concentrations

Levels of total IgG against TIV (Split Virion BP; Sanofi Pasteur MSD) or inactivated H1N1 were determined by ELISA as previously described (25, 29). Briefly, plasma samples were applied to Ag-coated ELISA plates (Nunc Maxisorp; Nunc) and bound IgG detected with goat anti-human IgG-peroxidase (Sigma-Aldrich) as the secondary Ab and SIGMAFAST OPD (Sigma-Aldrich) as the substrate. Ab levels were calculated as arbitrary ELISA units (AEU) with reference to values obtained for an in-house, high-titer standard plasma (assigned a value of 1000 AEU). The commercial AB serum used for tissue culture was estimated to contain 413.4 AEU against TIV and 273.7 AEU against H1N1, reflecting widespread natural exposure to influenza among the blood donor community. IgG-depleted AB plasma contained 7.6 and 14.6 AEU against TIV and H1N1, respectively. HCMV infection status was determined using a commercially available ELISA kit for anti-HCMV pp65 IgG (Biokit). Nineteen of the 52 study subjects (37%) were HCMV IgG seropositive.

### Abs and flow cytometric analysis

**FIGURE 1.**
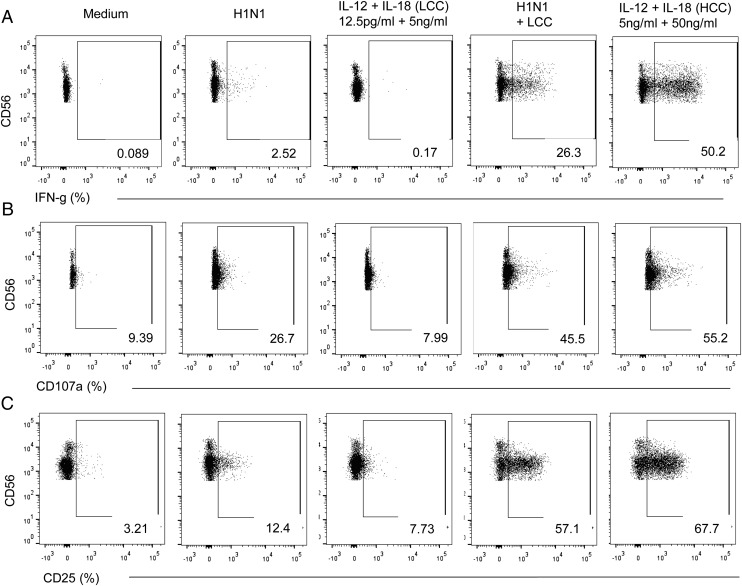
NK cell responses to influenza H1N1 virus. Flow cytometry plots are shown for NK cell IFN-γ (**A**), CD107a (**B**), and CD25 (**C**) responses to inactivated influenza virus in a representative individual 2 wk after vaccination. Responses were compared in gated NK cells cultured in, from left to right, medium alone, H1N1 Ag only, LCC (IL-12 12.5 pg/ml plus IL-18 10 ng/ml) alone, LCC plus H1N1, and HCC alone (IL-12 5 ng/ml plus IL-18 50 ng/ml). Percentages of total NK cells responding to the different stimuli are shown on each plot.

Phenotypic and functional analysis of ex vivo– and in vitro–stimulated NK cells was performed with the following mAbs: anti–CD3-V500 (clone UCHT1), anti–CD56-PECy7 (B159), anti–CD107a-FITC (H4A3) (all from BD Biosciences); anti–CD57-e450 (TB01), anti–CD16-allophycocyanin (CB16), anti–CD25-PerCP Cy5.5 (BC96) (all from eBioscience); or anti-CD71 FITC (OKT9; eBioscience), and anti-NKG2C PE (134591; R&D Systems). For in vitro assays, anti-CD107a Ab was added at the beginning of the culture. After staining for cell-surface markers, cells were fixed, permeabilized (Cytofix-Cytoperm; BD Biosciences), and washed (Perm-wash buffer; BD Biosciences) and either analyzed directly or stained with anti–IFN-γ–APC-eFluor 780–conjugated Ab (4S.B3; eBioscience). Ki67 staining (PerCP-eFluor 710–conjugated Ab; clone 20Raj1) was performed after inclusion of an APC-eFluor 780–conjugated fixable live-dead discriminatory dye with surface markers and subsequent fixation with Foxp3 intranuclear staining kit (all from eBioscience). Using the same protocols used for intracellular NK cell cytokine staining, T cells were labeled using anti–CD3-V500, CD56-PECy7, IL-2–allophycocyanin (MQ1 17-H12), IFN-γ–PE (B27) (all from BD Biosciences), CD4e450 (OKT4), and CD8-PECy5 (RPA-T8) (both from eBioscience). For ex vivo analysis, cells were additionally labeled with the following mAbs: anti–CD3-V500, anti–CD56-PECy7, and anti–CD94-FITC (HP-3D9) (BD Biosciences); anti–NKG2C-PE and anti–NKG2A-allophycocyanin (Z199; Beckman Coulter); and anti–CD8-PECy5, anti–CD57-e450, and anti–CD16-APC–eFluor 780 (eBioscience). Cells were acquired on an LSRII flow cytometer (BD Biosciences) using FACSDiva software. Data analysis was performed using FlowJo V10 (Tree Star). FACS gates set on unstimulated cells (medium alone or isotype controls) were applied across all samples and all conditions. Responses in which the gated subset contained <100 events were excluded. CD57^−^ subsets were gated using an isotype-matched control reagent (mIgG1–eFluor 450; eBioscience) and CD57^+^ subsets were gated according to a standardized mean fluorescence intensity (MFI) of 3000, with CD57^int^ cells lying between these populations. Sample gating strategies are shown in Figs. 1 and 8.

### Statistical analysis

Statistical analysis was performed using GraphPad Prism version 6.02 (GraphPad). Linear trends were evaluated using repeated-measures ANOVA. Functional responses between vaccination time points were compared using Wilcoxon signed-rank test, and intergroup comparisons between HCMV^+^ and HCMV-seronegative (HCMV^−^) individuals were performed using Mann–Whitney *U* test. Significance levels are assigned as *p < 0.05, **p < 0.01, ***p < 0.001, and ****p < 0.0001 for all tests.

## Results

### Potentiation of NK cell responses after influenza vaccination

To examine the effect of influenza vaccination on NK cell responses, we compared NK cell phenotype and function (by flow cytometry) among 52 healthy, previously unvaccinated, adult volunteers before (0; baseline) and 2, 4 and 12 wk after a single dose of seasonal TIV. Volunteers were randomly assigned to receive i.m. (*n* = 17), ID (*n* = 17), or IN (*n* = 18) vaccine. NK cell functional responses were assayed 18 h after in vitro restimulation to dissect both T cell–dependent and –independent responses. The route of vaccination had no significant effect on cellular immune responses, with enhancement of NK cell IFN-γ production and robust CD107a and CD25 expression being observed upon in vitro stimulation with influenza virus after vaccination via all three routes (data not shown). Data from all three arms of the study were therefore pooled for the majority of the analyses.

**FIGURE 2.**
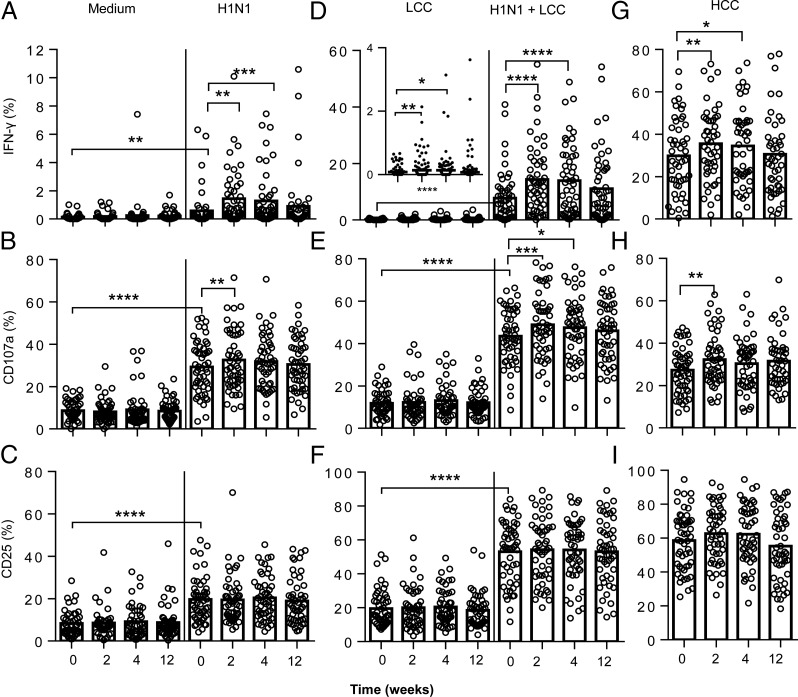
NK cell responses to influenza viruses are enhanced after influenza vaccination. PBMC were collected from 52 study subjects prior to (week 0) or after vaccination (weeks 2, 4, and 12) and cultured in vitro in culture medium alone, with vaccine strain influenza H1N1 (**A**–**C**) or with LCC (IL-12 12.5 pg/ml plus IL-18 10 ng/ml) alone or combined with H1N1 (**D**–**F**). Responses to HCC (IL-12 5 ng/ml + IL-18 50 ng/ml) were also determined before and after vaccination (**G**–**I**). Expression of IFN-γ (A, D, and G), CD107a (B, E, and H), and CD25 (C, F, and I) were analyzed within the total NK cell population. Paired statistical comparisons of NK cell responses before and after vaccination and between virus or cytokine responses and untreated cultures (medium) were performed using Wilcoxon signed-rank test. *p < 0.05, **p < 0.01, ***p < 0.001, ****p < 0.0001.

In vitro stimulation of PBMC with inactivated (vaccine strain) H1N1 virus induced significant upregulation of IFN-γ, CD25, and CD107a expression in baseline samples (H1N1 versus no Ag at week 0) (Figs. 1, 2A–C), and these responses were augmented in the presence of very low concentrations of IL-12 plus IL-18 (Figs. 1, 2D–F), suggesting limited induction of these cytokines by inactivated influenza virus in vitro. Moreover, IFN-γ responses to H1N1 were significantly enhanced 2 and 4 wk postvaccination in many, but not all, individuals: frequencies of IFN-γ^+^ cells were higher 2 wk postvaccination than before vaccination in 28 out of 52 subjects and higher 4 wk after vaccination than before vaccination in 20 out of 52 subjects. However, this effect had waned by 12 wk postvaccination (Figs. 1A, 2A). Postvaccination responses were further enhanced by very low concentrations of exogenous IL-12 plus IL-18 (LCC) such that enhancement of responses by vaccination was evident in 30 out of 52 individuals after 2 wk and 25 out of 52 individuals after 4 wk (Figs. 1A, 2D). NK cell CD107a responses against influenza H1N1 (Fig. 2B) and TIV (Supplemental Fig. 1) were high in baseline samples, reflecting high levels of anti-influenza Abs in the pooled human plasma used for culture (see *Materials and Methods*). There was a very modest effect of vaccination on CD107a responses at 2 wk postvaccination and no effect of vaccination on CD25 expression (Figs. 1B, 1C, 2B, 2C, 2E, 2F). Similar responses were observed after restimulation with whole TIV, albeit at lower frequency (Supplemental Fig. 1).

The modest (but statistically significant) increase in production of IFN-γ in response to low-dose IL-12 plus IL-18 observed 2 wk after influenza vaccination (Fig. 2D, *inset* graph) is highly reminiscent of the generation of memory-like NK cells in vitro with cytokine cocktails (8). To investigate this further, we tested whether influenza vaccination enhanced the response to higher concentrations of cytokines (in this case, 5 ng/ml IL-12 and 50 ng/ml IL-18) that were more in line with those used previously to cytokine-mediated human NK cell preactivation (Figs. 1, 2G–I) (8). Across the entire vaccination cohort, the proportions of NK cells producing IFN-γ and degranulating (i.e., expressing CD107a at the cell surface) in response to high concentrations of IL-12 plus IL-18 were significantly higher after vaccination, with increased frequencies compared with baseline values both 2 wk (29 out of 52 individuals) and 4 wk (32 out of 52 individuals) after influenza vaccination. Again, this effect had waned by 12 wk postvaccination (Fig. 2G, 2H). There was no effect of recent vaccination on the frequency of NK cells expressing after CD25 in vitro stimulation (Fig. 2I). Interestingly, in vitro restimulation with K562 cells also resulted in a small but significant increase in the frequencies of NK cells producing IFN-γ 4 wk postvaccination compared with baseline (Supplemental Fig. 2).

### Influenza virus–stimulated IFN-γ production postvaccination is dependent on IL-2, whereas CD107a expression requires IL-2 and immune complexes

**FIGURE 3.**
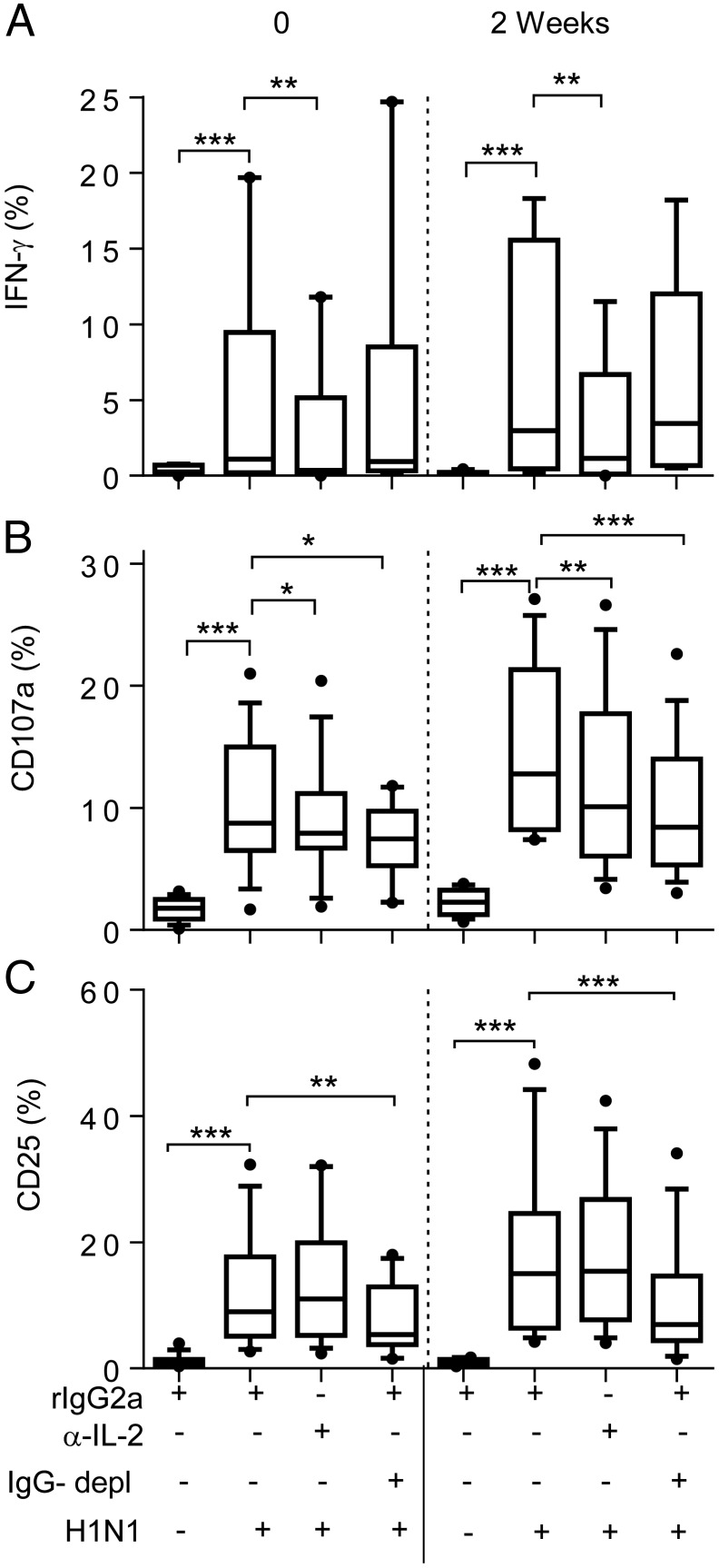
IL-2 and Ab dependence of postvaccination NK cell responses to influenza. PBMC, collected at baseline (0) or 2 wk after vaccination, were cultured in medium alone or with H1N1 influenza virus in the presence of anti-human IL-2 (or rat IgG2a isotype control) in either complete or IgG-depleted (IgG^−^) AB serum. IFN-γ (**A**), CD107a (**B**), and CD25 (**C**) responses were measured among total NK cells. Data are shown for 32 individuals (17 HCMV^−^ and 15 HCMV^+^) for whom sufficient cells were available at both time points. Boxes represent medians and interquartile ranges, whiskers represent 90th percentiles, and outliers are shown by solid dots. Paired comparisons between treatments were performed using Wilcoxon signed-rank test. *p < 0.05, **p < 0.01, ***p < 0.001.

We next investigated whether, in addition to the intrinsic effects of vaccination on NK cell cytokine responsiveness, enhancement of NK cell IFN-γ and CD107a responses to H1N1 virus postvaccination also depended on Ag-specific T cells and plasma IgG. Assays were carried out with whole (inactivated) H1N1 virus using mixed PBMC (containing detectable influenza-specific IL-2^+^CD4^+^ T cells; Supplemental Fig. 3) and pooled human AB serum containing anti-H1N1 Abs to standardize the level of influenza-specific IgG in cultures (see *Materials and Methods*) (Fig. 3). IL-2 blockade with a neutralizing anti–IL-2 mAb significantly reduced NK cell IFN-γ (Fig. 3A) and CD107a (Fig. 3B) responses both at baseline and 2 wk postvaccination compared with the isotype-matched control. IL-2 neutralization had no significant effect on CD25 upregulation (Fig. 3C). To determine whether anti-influenza Abs also played a role in NK activation, PBMCs were cultured in either intact AB serum (i.e., containing influenza-specific IgG) or in AB serum from which IgG had been depleted. IgG depletion had no effect on IFN-γ responses (Fig. 3A) but significantly reduced both CD107a (Fig. 3B) and CD25 (Fig. 3C) responses in both baseline and 2-wk postvaccination samples.

Overall, these data indicate an important role for IL-2 in H1N1-induced NK cell IFN-γ responses both in prevaccination responses and in the enhanced response resulting from vaccination.

In contrast, optimal CD107a responses were dependent on both IL-2 and Ab/immune complexes, consistent with IL-2–mediated postvaccination enhancement of CD107a responses to H1N1 in PBMC assays in which anti-influenza IgG levels (in AB serum) are held constant (Fig. 1B).

### Vaccination does not augment Ab-dependent NK cell responses

**FIGURE 4.**
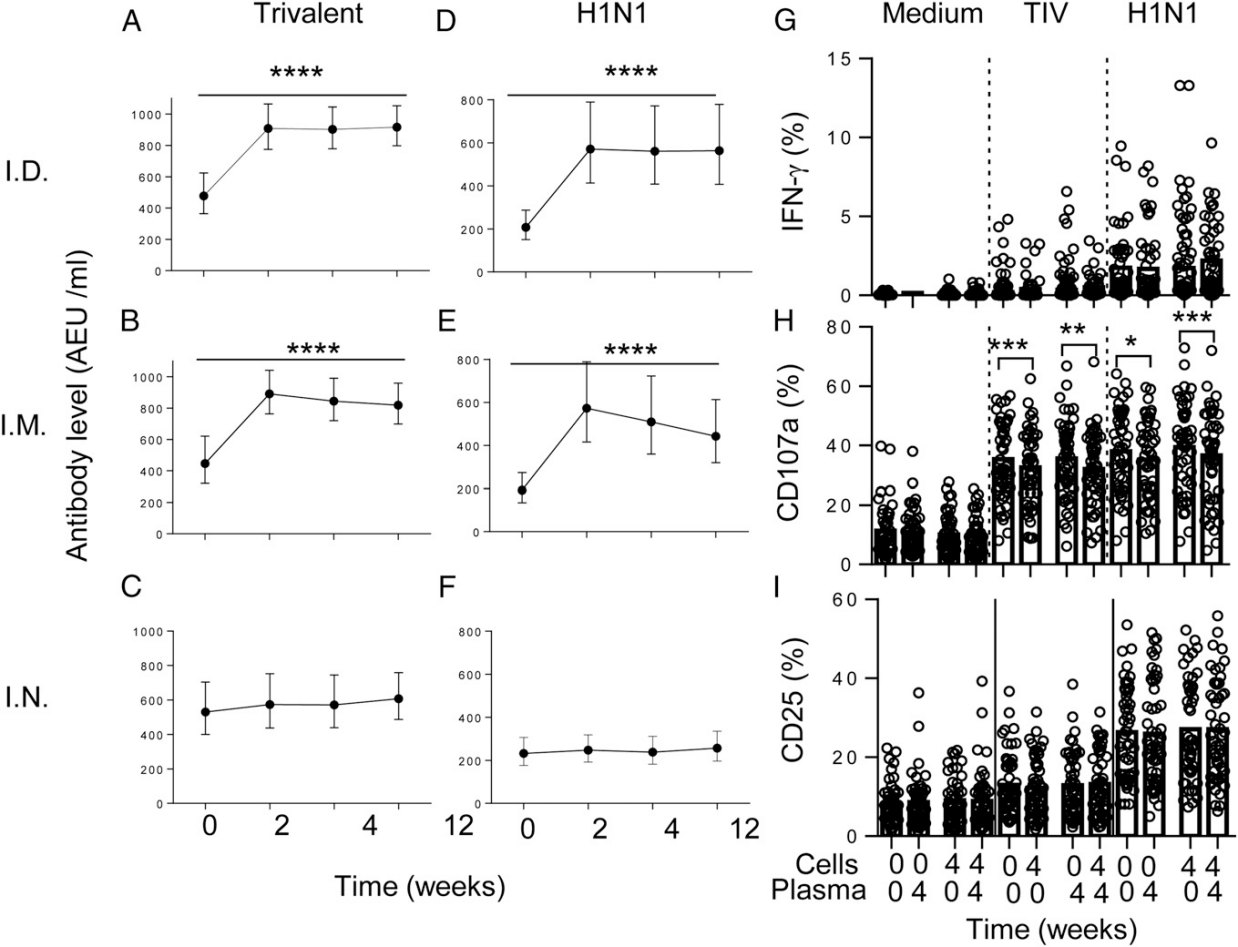
Boosting of anti-influenza IgG by vaccination does not enhance NK cell responses. Increased levels of serum IgG against TIV (**A**–**C**) or H1N1 (**D**–**F**) were detected after vaccination with ID (A and D) and i.m. (B and E) but not after IN (C and F) vaccination. Data are shown as median values with IQRs. Expression of IFN-γ (**G**), CD107a (**H**), and CD25 (**I**) was measured in NK cells from all 52 study donors at baseline (0) or 4 wk postvaccination (4) after culture with inactivated H1N1 virus in the presence of autologous plasma taken either at baseline (0) or 4 wk after vaccination (4). Trend analysis was performed using one-way ANOVA with correction for multiple comparisons. Paired comparisons were made using Wilcoxon signed-rank test. *p < 0.05, **p < 0.01, ***p < 0.001, ****p < 0.0001.

In view of the role of serum Ab in the induction of NK cell CD107a and CD25 responses to influenza virus, we assessed the impact of increasing autologous plasma Ab concentrations after vaccination on the induction of NK cell responses. Plasma IgG levels to TIV and H1N1 were assessed at baseline, 2, 4, and 12 wk postvaccination and compared by route of vaccination and HCMV infection status (Fig. 4). All of the donors recruited to this study were seropositive for IgG to TIV at baseline, and 46 out of 52 were seropositive for H1N1, indicating widespread prior exposure to seasonal influenza. Intramuscular or ID vaccination with inactivated TIV boosted pre-existing concentrations of IgG to both TIV and H1N1, in both HCMV^+^ and HCMV^−^ individuals, but there was only a very modest systemic IgG response to the live, attenuated IN vaccine (Fig. 4A–F), in line with previously published data on IN vaccination (30).

To determine whether boosting of anti-influenza Ab titers by vaccination also boosted NK cell responses, baseline or postvaccination PBMC were cultured with TIV or H1N1 virus in medium containing autologous plasma taken either at baseline or 4 wk after vaccination. No effect was observed of postvaccination plasma on IFN-γ (Fig. 4G) or CD25 (Fig. 4I) responses. Surprisingly, CD107a responses were significantly reduced when cells were cultured in postvaccination plasma (Fig. 4H).

These data indicate that pre-existing concentrations of anti-influenza Abs are sufficient to support NK cell degranulation and CD25 induction in response to influenza virus in individuals with prior exposure to seasonal influenza virus strains.

### In vitro preactivation mimics the effects of vaccination

**FIGURE 5.**
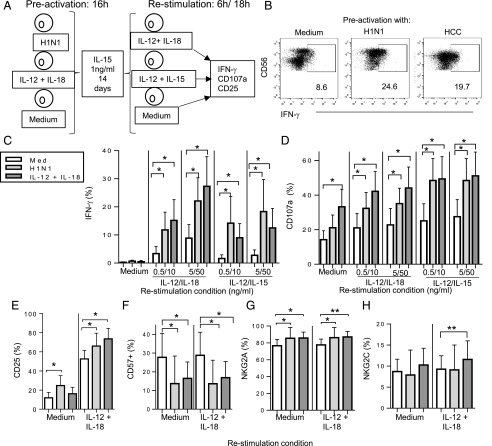
Influenza virus induces memory-like NK cells in vitro. (**A**) An in vitro model for memory-like NK cell generation was used to test whether influenza H1N1 or HCC can preactivate NK cells for enhanced responsiveness after 2 wk of culture. PBMC were initially cultured for 16 h in medium alone, with inactivated influenza H1N1 or with HCC. All cultures contained 1 ng/ml IL-15 to ensure NK cell survival. Cultures were then washed extensively and maintained in medium (containing 1 ng/ml IL-15) for 14 d. After further washes, cells were restimulated in vitro with varying concentrations of IL-12 combined with either IL-15 or IL-18. NK cell responses were monitored after 6 or 18 h. (**B**) Representative flow cytometry plots for IFN-γ production after HCC (IL-12 5 ng/ml plus IL-18 50 ng/ml) restimulation of control NK cells (medium alone, no preactivation) or NK cells preactivated with H1N1 or HCC. Enhancement of IFN-γ production (**C**), CD107a expression (**D**), and CD25 expression (**E**) in eight individuals after NK cell preactivation with H1N1 (light gray bars) and HCC (dark gray bars) compared with no preactivation (open bars). Cells were restimulated with the indicated concentrations of IL-12 combined with either IL-15 or IL-18. Frequencies of NK cells expressing CD57 (**F**), NKG2A (**G**), and NKG2C (**H**) were determined in preactivated NK cells cultured in medium alone or restimulated with IL-12 plus IL-18. Comparisons between culture conditions were made using Wilcoxon signed-rank test. *p < 0.05, **p < 0.01.

In view of the enhanced NK cell response to IL-12 plus IL-18 after vaccination (Fig. 2), we investigated whether influenza virus H1N1 could preactivate NK cells in vitro and, if so, whether this was cytokine mediated. Using an established protocol (8), PBMC were incubated with inactivated influenza H1N1 virus or, as a positive control, HCC (IL-12 5 ng/ml plus IL-18 50 ng/ml) for 16 h in the presence of a low concentration of IL-15 (1 ng/ml); IL-15 alone provided a negative control (Fig. 5A). The cells were washed (to remove the stimuli), maintained in IL-15 for 2 wk, and then restimulated with varying concentrations of either IL-12 plus IL-18 or IL-12 plus IL-15 for 6 h (Fig. 5A). Both IFN-γ and CD107a responses were higher in NK cells that had been preactivated in vitro with H1N1 than in NK cells maintained in IL-15 alone, and these responses were broadly similar to those induced by preactivation with high concentrations of IL-12 and IL-18 (Fig. 5B–D). Preactivation with H1N1 or HCC had a modest but statistically significant effect on CD25 expression in response to IL-12 plus IL-18, but this was only evident after 18 h of cytokine restimulation (Fig. 5E) rather than 6 h (data not shown). Of interest, the proportion of CD57^+^ NK cells was lower in preactivated cultures than in cultures maintained in IL-15 alone (Fig. 5F), and there was a modest but statistically significant increase in the proportion of NKG2A^+^ (but not NKG2C^+^) cells (Fig. 5G, 5H).

### Type 1 IFNs contribute to NK cell preactivation in vitro

**FIGURE 6.**
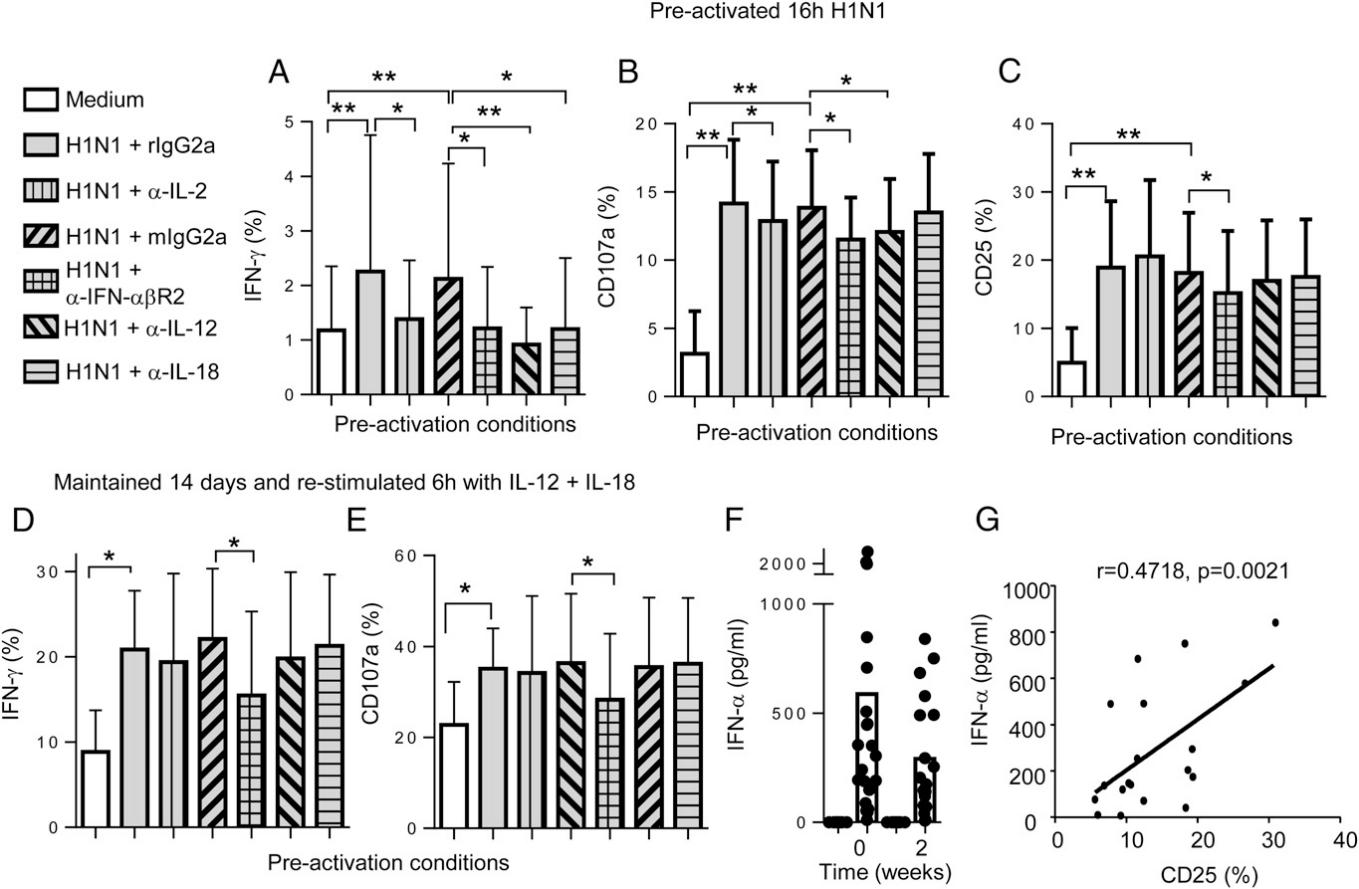
IFN-αβR2 blockade abrogates in vitro preactivation of NK cells by influenza virus (**A**–**E**). PBMCs from eight individuals were preactivated with H1N1 in the presence of neutralizing anti–IL-2, anti–IL-12, anti–IL-18, or blocking anti–IFN-αβR2 Ab or rat or mouse isotype control reagents, as indicated. The frequencies of NK cells expressing IFN-γ (A and D), CD107a (B and E), or CD25 (C) after 6-h restimulation with HCC (IL-12 5 ng/ml plus IL-18 50 ng/ml) were measured directly (A–C) or after maintenance in IL-15 replete medium for 14 d (D and E). (**F**) IFN-α concentrations in culture supernatants of H1N1-stimulated PBMCs from 32 individuals (17 HCMV^−^, 15 HCMV^+^) before (0) and 2 wk after vaccination. (**G**) Correlation between H1N1-induced IFN-α concentration and H1N1-induced CD25 expression 2 wk postvaccination. Statistical comparisons between NK cells with and without preactivation or between different neutralizing/blocking conditions were made by Mann–Whitney *U* test. *p < 0.05, **p < 0.01.

To determine whether, and if so which, cytokines were involved in NK cell preactivation by H1N1 virus, the 16-h preactivation experiments were repeated in the presence or absence of neutralizing mAbs to IL-2, IL-12, or IL-18 or a blocking Ab to IFN-αβR2, and NK cells were immediately tested for IFN-γ, CD107a, and CD25 expression (Fig. 6); isotype-matched Abs with irrelevant specificity were used as negative controls. All four neutralizing or blocking Abs significantly reduced IFN-γ production by H1N1-activated NK cells (Fig. 6A). Similar but more limited effects were observed for CD107a expression (Fig. 6B) but only anti–IFN-αβR2 significantly reduced CD25 expression (Fig. 6C).This experiment was then repeated, with neutralizing Abs present at the time of preactivation, on cells that had then been rested in IL-15 for 14 d and restimulated. In this case, however, blockade of IFN-αβR2 significantly reduced both the IFN-γ (Fig. 6D) and the CD107a (Fig. 6E) response to IL-12 plus IL-18, but neutralization of the other cytokines had no significant effect.

To explore the hypothesis that type 1 IFNs induced in the PBMC cultures by H1N1 virus were required for NK cell preactivation, we assessed IFN-α and IFN-β secretion in culture supernatants of H1N1-stimulated PBMC collected from study subjects at baseline (before vaccination) and 2 wk after vaccination. IFN-α (at concentrations in excess of 100 pg/ml) was detected in cell supernatants from the majority of study subjects at both time points (Fig. 6F), but IFN-β was not detected (data not shown). Moreover, a possible role for IFN-α in the upregulation of CD25 on NK cells is supported by a direct correlation between the frequency of CD25^+^ cells and the level of IFN-α in supernatants (Fig. 6G).

### HCMV exposure influences NK cell responses after vaccination

Our previous cross-sectional studies have suggested that NK cells from HCMV-uninfected individuals make superior IFN-γ and CD25 responses to vaccine Ags in vitro, in part relating to higher frequencies of less well-differentiated CD57^−^ NK cell subsets (26, 31). As HCMV infection promotes expansion of CD57^+^ NK cell subsets at the expense of CD57^−^ subsets (28, 32–34), we reasoned that vaccine-induced generation of memory-like NK cells could differ between HCMV-infected (HCMV^+^) and HCMV-uninfected (HCMV^−^) subjects.

**FIGURE 7.**
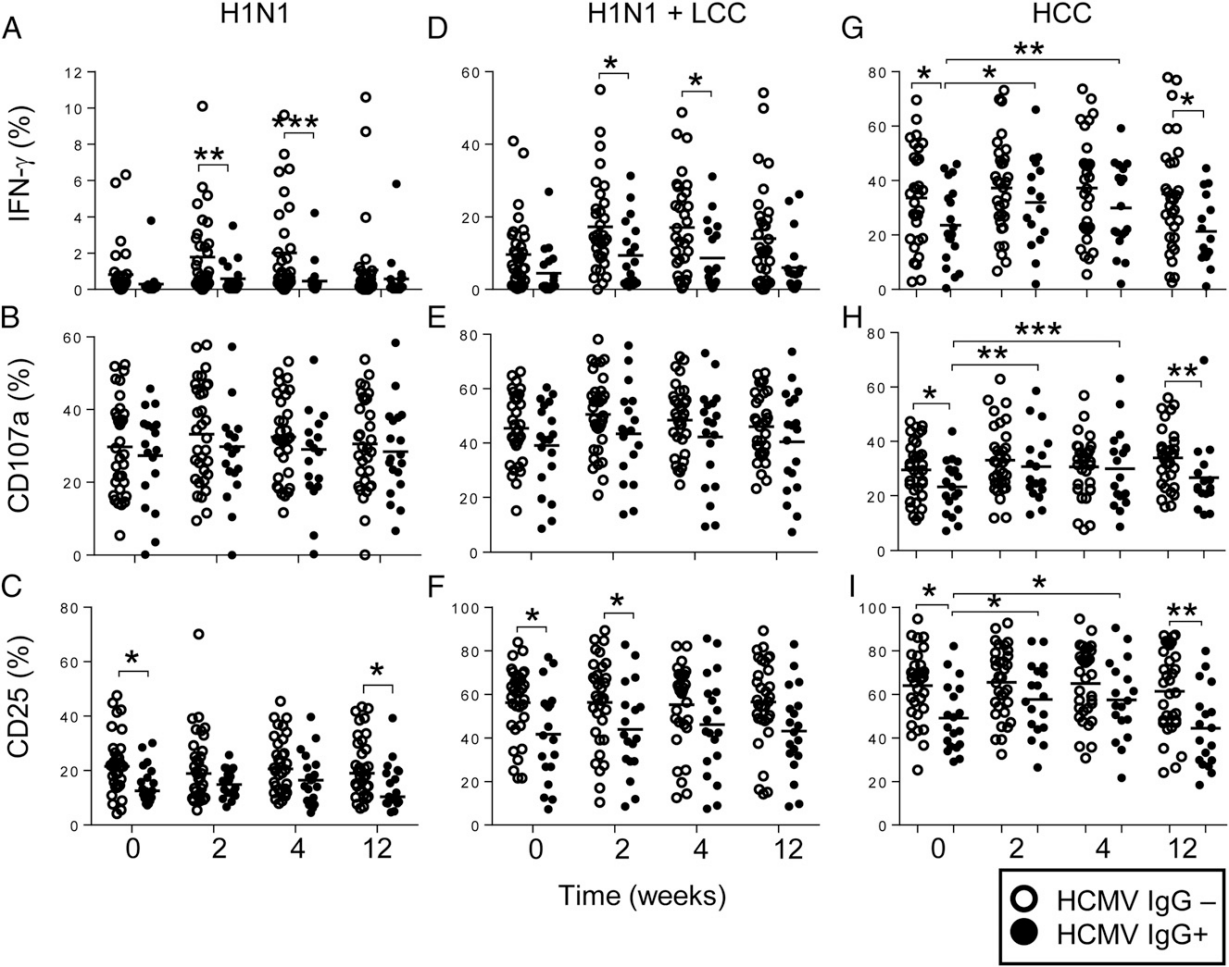
Impact of HCMV infection status on postvaccination NK cell responses. NK cell IFN-γ, CD107a, and CD25 responses are shown for 33 HCMV IgG-seronegative (open symbol) and 19 HCMV IgG-seropositive individuals (closed symbol) after stimulation with H1N1 virus alone (**A**–**C**), H1N1 in the presence of LCC (**D**–**F**), or HCC (**G**–**I**) before (0) or 2, 4, or 12 wk after vaccination. Bars represent mean values. Comparisons between HCMV^−^ and HCMV^+^ individuals were made using Mann–Whitney *U* tests. *p < 0.05, **p < 0.01, ***p < 0.001.

There were marked and highly significant differences between HCMV^−^ and HCMV^+^ subjects in their NK cell responses to influenza vaccination (Fig. 7). NK cell IFN-γ responses to H1N1 and to H1N1 plus LCC were robustly enhanced among HCMV^−^ individuals, but there was limited or no augmentation of the IFN-γ response among HCMV^+^ subjects (Fig. 7A, 7D). Although CD107a responses did not differ between HCMV^+^ and HCMV^−^ subjects (Fig. 7B, 7E), upregulation of CD25 was significantly greater among HCMV^−^ than HCMV^+^ subjects both at baseline and after vaccination (Fig. 7C, 7F). Moreover, CD25 upregulation by H1N1 stimulation, as measured by MFI, was significantly higher among HCMV^−^ individuals than among HCMV^+^ individuals across the entire vaccination time course (baseline: HCMV^−^, median MFI 529, interquartile range [IQR] 477–627; HCMV^+^, median MFI 451, IQR 404–572, *p* = 0.01; week 2: HCMV^−^, median 550, IQR 496–636; HCMV^+^, median 489, IQR 426–552, *p* = 0.008), and similar effects were observed after stimulation with H1N1 plus LCC (data not shown). In vitro stimulation with TIV also resulted in stronger IFN-γ and CD25 responses in HCMV^−^ individuals than in HCMV^+^ individuals (Supplemental Fig. 2).

However, somewhat unexpectedly, when we stratified the data by HCMV infection status, postvaccination enhancement of NK cell IFN-γ responses to high concentrations of IL-12 plus IL-18 was observed only among HCMV^+^ individuals, in whom the frequencies of responding cells approached those consistently observed in HCMV^−^ individuals (Fig. 7G). Stratification by HCMV serostatus also revealed significant increases postvaccination in CD107a and CD25 expression (Fig. 7H, 7I). Enhancement of CD107a, CD25 expression, and IFN-γ production among HCMV^+^ subjects was evident at 2 and 4 wk postvaccination but had waned by 12 wk (Fig. 7G–I). Differences in the proportions of CD25^+^ NK cells were mirrored in the MFI of CD25 expression, with significantly increased MFI for CD25 in HCC-stimulated NK cells from HCMV^+^ individuals 2 wk postvaccination (baseline: median 849, IQR 745–1061; week 2: median 1014, IQR 841–1147, *p* = 0.003).

### NK cell differentiation status affects vaccine-induced responses

**FIGURE 8.**
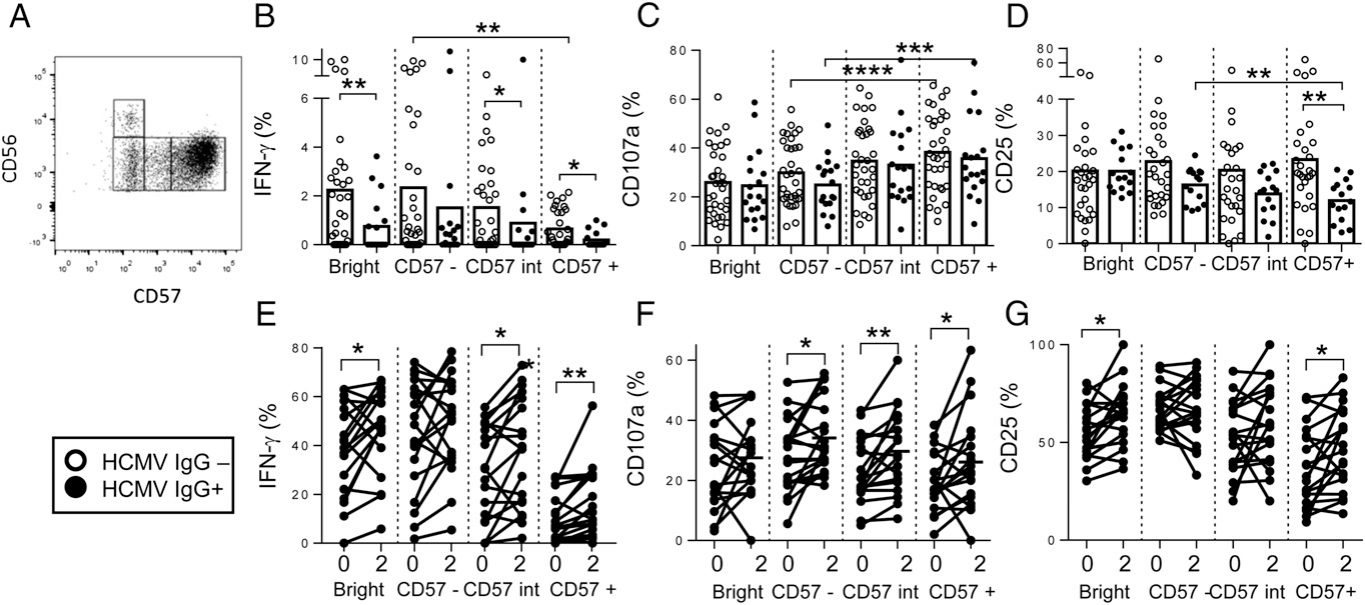
Responses of CD57-defined NK cell subsets and impact of HCMV exposure. (**A**) Gating strategy for CD57-defined NK cell subsets. PBMCs were restimulated with H1N1 and IFN-γ (**B** and **E**), CD107a (**C** and **F**), and CD25 (**D** and **G**) responses were analyzed for each CD56/CD57-defined NK cell subset. (B–D) Responses for cells collected 2 wk after vaccination are stratified by HCMV infection status: open circles, HCMV^−^ (*n* = 33) individuals; closed circles, HCMV^+^ (*n* = 19) individuals. Bars represent mean values. Significant values for trends across CD56^dim^ NK cell subsets (repeated-measures ANOVA) and comparisons between HCMV^−^ and HCMV^+^ individuals (Mann–Whitney *U* test) are shown. (E–G) For each subset, baseline (0) responses are compared with postvaccination (2 wk) by Wilcoxon signed-rank test; paired data for each individual are joined by a solid line. *p < 0.05, **p < 0.01, ***p < 0.001, ****p < 0.0001.

Cross-sectional studies in adults of NK cell responses to childhood vaccine Ags have revealed that IFN-γ responses are more or less restricted to the CD56^bright^ and CD56^dim^CD57^−^ NK cell subsets but that degranulation responses are retained or enhanced among CD57^+^ NK cells (25, 26). As HCMV infection leads to expansion of the CD57^+^ NK cell subset, reduced postvaccination IFN-γ responses among HCMV^+^ subjects may be due simply to differences in subset distribution. NK cell responses to H1N1 virus were therefore analyzed according to their expression of CD57 (Fig. 8).

Stratification of NK cell responses to H1N1 influenza by CD57 expression (see Fig. 8A for gating strategy) in HCMV^+^ and HCMV^−^ individuals revealed that IFN-γ responses were lower within each subset in HCMV^+^ individuals than in HCMV^−^ individuals. Data for the 2-wk postvaccination time point are shown in Fig. 8B, with similar effects evident at all other time points (data not shown). This is consistent with previous data suggesting that the effects of HCMV infection go beyond a simple redistribution of NK cell subsets (25). The frequencies of NK cells expressing CD107a upon stimulation with H1N1 virus increased significantly with increasing CD57 expression 2 wk after vaccination but did not differ between HCMV^+^ and HCMV^−^ individuals (Fig. 8C). Importantly, CD25 induction by H1N1 in HCMV^−^ subjects did not differ significantly by subset, whereas among HCMV^+^ subjects CD25 induction declined with increasing CD57 expression and was significantly lower among HCMV^+^ than HCMV^−^ donors in the CD57^+^ subset (Fig. 8D).

As CD56^bright^ and CD56^dim^CD57^−^ NK subsets are enriched for cells expressing cytokine receptors, we next analyzed whether enhancement of IL-12 plus IL-18 responsiveness in HCMV^+^ individuals was due to preferential effects on these subsets (Fig. 8E–G). As expected, CD56^bright^ and CD56^dim^CD57^−^ NK subsets from both HCMV^−^ and HCMV^+^ individuals contained higher frequencies of IFN-γ and CD25-expressing cells compared with the CD57^+^ subset in response to IL-12 plus IL-18 (Fig. 8E, 8G). However, in HCMV^+^ individuals, enhancement of these responses after vaccination was observed across NK cell subsets, and CD107a expression was significantly enhanced within all CD56^dim^CD57-defined populations (Fig. 8F). Little or no effect was observed on NK cell subset responses of HCMV^−^ individuals to HCC (data not shown). These data indicate that HCC can overcome a high activation threshold for NK cell CD25 and IFN-γ expression in vaccine-preactivated NK cells from HCMV^+^ individuals.

### NK cell phenotype is modified by influenza vaccination according to HCMV exposure

**FIGURE 9.**
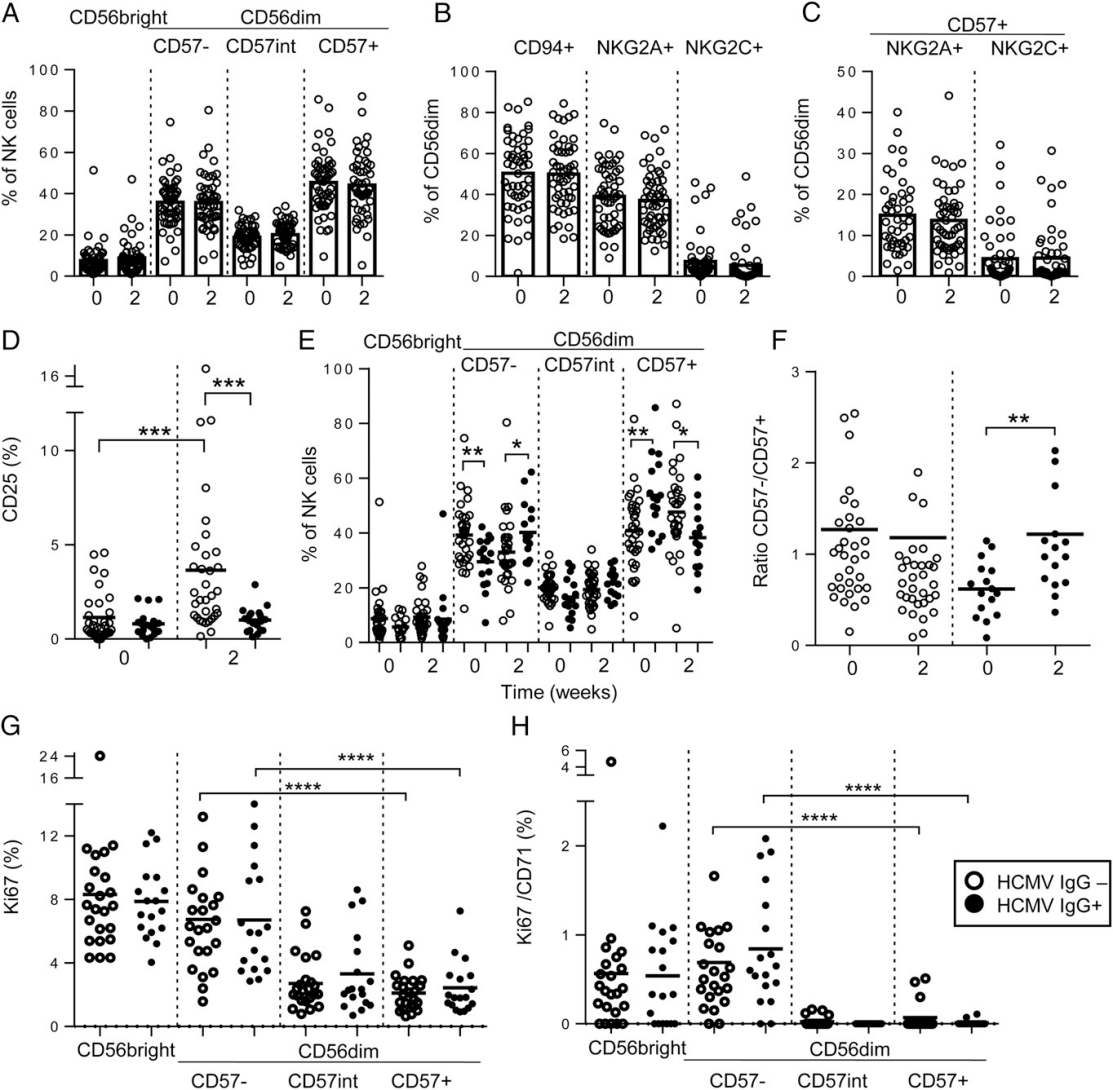
Impact of influenza vaccination and HCMV infection status on ex vivo NK cell-surface phenotype. Frequencies of CD56^bright^ and CD56^dim^CD57-defined subsets (**A**), CD94-, NKG2A-, and NKG2C-expressing NK cells (**B**), and NKG2A^+^CD57^+^ or NKG2C^+^CD57^+^ NK cells (**C**) were measured directly ex vivo before (0) or 2 wk after vaccination for all 52 study subjects. NK cells from HCMV^−^ (open circles; *n* = 33) and HCMV^+^ (closed circles; *n* = 19) subjects were compared for ex vivo frequencies of CD25^+^ cells (**D**), proportions of cells in each subset (**E**), and the ratio of CD56^dim^CD57^−^ to CD56^dim^CD57^+^ cells before (0) or 2 wk after vaccination (**F**) and for frequencies of NK cells in each subset expressing Ki67 (**G**) or Ki67 in combination with CD71 (**H**) 2 wk after vaccination. Bars represent mean values. Mann–Whitney *U* test was used for intergroup comparisons, and trend analysis was performed using a one-way ANOVA with correction for repeated measures. ** *p* < 0.01, ***p < 0.001, ****p < 0.0001.

Although influenza vaccination influenced cytokine-driven responses across all CD57-defined NK cell subsets in HCMV^+^ individuals, this did not rule out the possibility that vaccination could induce global changes in the frequencies of functional NK cell subpopulations, as seen in the in vitro experiments described above (Fig. 5). To determine whether the effects of influenza vaccination on NK cell responses were attributable to gross changes in NK cell phenotype in vivo, samples from HCMV^+^ and HCMV^−^ individuals were analyzed directly ex vivo (i.e., without in vitro restimulation) (Fig. 9). There was no evidence of any change in the ex vivo distribution of CD56/CD57/NKG2C or CD56/CD57/NKG2A-defined NK cell subsets after vaccination (Fig. 9A–C), and, in contrast to previous studies (35), we did not see any change in NKp46 expression (data not shown). Among HCMV^−^ subjects, the proportion of NK cells expressing CD25 (IL-2Rα) ex vivo was significantly higher 2 wk after vaccination than at baseline, whereas this effect was not seen in NK cells from HCMV^+^ subjects (Fig. 9D), and no significant increase in ex vivo expression of IFN-γ or CD107a was detected at this or subsequent time points (data not shown). These data are consistent with studies previously reporting CD25 being a marker of NK cell activation at early time points after influenza vaccination (22).

As expected, the proportion of CD57^+^ NK cells was higher at baseline among HCMV^+^ subjects than HCMV^−^ subjects, and the proportion of CD57^−^ NK cells was lower (Fig. 9E) (25, 32, 33). However, among HCMV^+^ individuals, the proportions of CD57^−^ NK cells increased noticeably after vaccination (Fig. 9E), resulting in a significant increase in the ratio of CD57^−^ to CD57^+^ NK cells, which was not seen in HCMV^−^ subjects (Fig. 9F). Expression of Ki67, and coexpression of Ki67 with CD71 (the transferrin receptor), on a significant proportion of both CD56^bright^ and CD56^dim^CD57^−^ NK cells 2 wk after vaccination (Fig. 9G, 9H) is indicative of proliferation (recent cell division) within the CD57^−^ NK cell subset. These data are consistent with vaccination promoting proliferation of less differentiated, cytokine-responsive NK cells and normalization of frequencies of these cells between HCMV^−^ and HCMV^+^ individuals, leading to improved responsiveness to exogenous cytokines.

## Discussion

Training of lymphoid and myeloid cells upon pathogen exposure enables them to mount superior responses upon subsequent exposure (1, 2). In this study, we demonstrate that the generation of memory-like NK cells, observed with recombinant cytokines in vitro (8, 20), also occurs in vivo after vaccination with either live attenuated or killed influenza virus. NK cells induced by influenza vaccination share all the characteristics of cytokine preactivated memory-like (7) NK cells, including long-term enhancement (for at least a month) of IFN-γ responses to innate cytokines and MHC class I–deficient target cells and a role for CD25 upregulation and IL-2 in enhancing these responses (8, 11, 20). Taken together, our data support a model for generation of memory-like NK cells by influenza virus involving upregulation of CD25 by virus-induced IFN-α; selective, IL-2–driven expansion of less differentiated (CD57^−^) NK cells; rapid and enhanced responsiveness of these cells to subsequent cytokine stimulation; and, in the absence of further boosting or infection, a resting or reversion phase (Supplemental Fig. 4).

Our in vitro model of long-term NK cell preactivation by H1N1 influenza demonstrates a role for IFN-αβR2 in virus-induced preactivation. This is consistent with studies in IFN-αR^−/−^ mice that point to a role for IFN-α in murine CMV- (MCMV)–driven NK cell expansion and memory cell formation, in this case by prevention of fratricidal killing (36), and for type I IFNs (but not IL-12 and IL-18) in induction of NK cell IFN-γ and degranulation in a murine model of influenza virus infection (37). A role for IFN-α in preactivation of human NK cells by influenza virus is supported by finding biologically relevant concentrations of IFN-α in H1N1-stimulated PBMC cultures and is consistent with reports of raised IFN-α concentrations in human plasma up to 7 d after influenza vaccination (22). The effect of IFN-αβR2 blockade was, however, partial, raising the possibility that additional myeloid lineage-derived cytokines contribute to influenza virus–induced memory-like NK cells. Indeed, consistent with previous studies, we observed some contribution of IL-12 (and IL-18) to IFN-γ production during the prestimulation phase in vitro (38). Additionally, although our in vitro studies employed inactivated vaccine Ag, a role for NK cell receptor–ligand interactions or membrane-bound cytokines, as shown in other systems, has not been excluded (38, 39). Further studies combining neutralizing/blocking reagents will clarify whether synergies between influenza induced cytokines are required for memory-like NK cell generation.

Preactivation and memory-like NK cell generation by H1N1 influenza in vitro also demonstrates a marked acceleration of the NK cell IFN-γ response, with IFN-γ^+^ cells detected as early as 6 h after restimulation, rather than the 16–18-h response typically observed for primary in vitro stimulation. This suggests that preactivated NK cells may be able to respond more rapidly in vivo during infection. Similarly accelerated responses are observed in NK cells preactivated with high concentrations of cytokine (8). This accelerated effector response may, however, be independent of the IL-2/CD25-driven maintenance of cytokine-responsive CD57^−^ NK cells that we also observed: CD25 upregulation occurred significantly later (18 h) than the IFN-γ response and would also require IL-2 release from Ag-specific T cells. Cytokine-induced preactivation of NK cells may therefore comprise two distinct phases: an initial phase in which IL-12/IL-18–induced demethylation of the *IFNG* locus enhances and accelerates IFN-γ production (27) and a subsequent phase in which IFN-α–induced upregulation of CD25 facilitates IL-2–dependent proliferation of CD57^−^ NK cell subsets.

Experiments in which we depleted IgG from NK cell cultures also revealed a potential contribution of Ab–Ag immune complexes to CD25 upregulation, indicating that FcR-mediated signals transduced by influenza-specific Abs and Ag may synergize with innate cytokines to enhance NK cell responses. Notably, however, there was sufficient influenza-specific IgG present in plasma of unvaccinated subjects at baseline to support these responses, indicating that, at least in United Kingdom adults, natural exposure to influenza induces sufficient Ab to mediate Ab-dependent cytotoxic responses and that this response is not noticeably enhanced by vaccination. It remains to be seen whether mucosal vaccine-induced IgA can augment responses of Fcα^+^ NK cells to influenza infection (30, 40).

Our studies reveal a redistribution of NK cell subsets according to their differentiation stage after both H1N1 and cytokine preactivation of NK cells in vitro and in NK cells sampled ex vivo before and after vaccination. In vitro priming with H1N1 clearly favors enrichment of cells lacking the late differentiation marker CD57. One caveat of our in vitro model is the potential for preferential activation and maintenance of CD56^bright^CD57^−^CD94^+^ cells, with higher sensitivity for cytokines, within influenza-stimulated cultures. However, we observed no overall change in the frequency and CD56^bright^ NK cells ex vivo after influenza vaccination, whereas significant redistribution occurred within CD56^dim^ NK cells. This was particularly evident in HCMV^+^ individuals, with the effect that 2 wk after vaccination, the ratio of CD57^−^ to CD57^+^ cells in HCMV^+^ subjects has been restored to levels seen in HCMV^−^ subjects. The increased frequencies of Ki67^+^ and CD71^+^CD56^dim^CD57^−^ NK cells that we observed ex vivo after influenza vaccination are, however, consistent with proliferative expansion of CD56^dim^CD57^−^ cells and reminiscent of data on yellow fever vaccination in which CD57-defined subset redistribution correlated strongly with increased Ki67 expression (41). These data thus confirm and extend observations made previously for cytokine preactivated NK cells (8) and suggest that any benefits of cytokine-mediated preactivation may be particularly apparent in HCMV^+^ individuals.

This study reveals important differences between NK cell responses to influenza Ags and those driven by high concentrations of exogenous cytokines, which may underlie the differential responsiveness in HCMV^−^ and HCMV^+^ individuals. Although vaccine-mediated preactivation may play a role in boosting NK cell responses to influenza virus both in HCMV^−^ and HCMV^+^ subjects, lower CD25 expression in HCMV^+^ individuals (particularly on late differentiated NK cells) could limit the impact of T cell–derived IL-2 in costimulation and maintenance of memory-like NK cells. Lower frequencies of CD25 expressing NK cells in HCMV-infected subjects after H1N1 stimulation may be partially attributed to reduced IFN-α production because median concentrations of IFN-α in H1N1 culture supernatants were marginally lower among HCMV^+^ individuals than among HCMV^−^ individuals (baseline: 401.0 and 194.3 pg/ml, respectively; 2 wk postvaccination: 214.8 and 141.7 pg/ml, respectively), but these differences did not reach statistical significance. In contrast, cells from HCMV^+^ donors responded much less well to IL-12 and IL-18 compared HCMV^−^ individuals, but this defect was partially overcome after vaccination.

The longevity of memory-like NK cells may well be linked to the duration of the underlying stimulus, being longer lasting when induced by a virulent, replicating virus such as hantavirus or yellow fever virus (41, 42) than when induced by vaccination (as in this study) or by exogenous cytokines (7, 8). The use of an inactivated vaccine in our studies, although not completely ruling out the possibility of the persistence of residual Ag, supports a model in which cytokine preactivation outlasts clearance of Ag. Our observation of similar levels of NK cell preactivation induced by mucosal, dermal, and i.m. vaccination supports a role for common, systemically produced factors. Intrinsic changes affecting cytokine responsiveness, including epigenetic changes, combined with upregulation of CD25, are likely to be involved in the induction and maintenance of memory-like NK cells, particularly those that are relatively undifferentiated.

Our data, and that of others, reveal differences between the global effects of cytokine-induction of memory-like NK cells and more specific, receptor-mediated, ITIM-dependent generation of memory NK cells. Ligation of murine Ly49H by MCMV m157 generates Ly49H^+^ NK cells with long-term enhanced responsiveness to MCMV. In humans, NK cells expressing NKG2C or activating killer Ig-like receptor expand in response to HCMV-infected fibroblasts in vitro and adaptive expansions of NKG2C^+^ cells (as well as NKG2C^−^ cells lacking the FcεRγ signaling adaptor) are evident in the blood of HCMV^+^ individuals (43–47). By contrast, we found no evidence that cytokine-mediated generation of memory-like NK cells after vaccination favored particular NKG2A- or NKG2C-defined NK cell subsets.

Although the pathways involved in the generation of cytokine-induced memory-like NK- and ITIM-generated memory NK cells may be distinct, it is likely that cytokine-driven mechanisms can impact the latter populations. NK cells of HCMV^+^ individuals may be skewed toward CD57^+^ Ab-dependent effectors, but these individuals nevertheless retain the capacity to expand a population of highly cytokine-responsive and cytokine-producing cells in situations (such as acute infection, mimicked in this case by vaccination) when this would be advantageous: cytokine responsiveness of CD57^+^ NK cells was enhanced after vaccination. Furthermore, several studies indicate a role for cytokines in the regulation of NK cells that have been expanded by interactions with infected target cells. IL-12 in particular is essential for induction of CD25 and for generation and maintenance of memory-like NK cells in MCMV-infected mice (48, 49) and for upregulation of CD25 on NKG2C^+^ NK cells in response to HCMV/HLA-E (45). In addition, enhanced cytokine responsiveness in NKG2C^bright^ NK cells may be facilitated by epigenetic reprogramming of the IFN-γ locus after specific receptor–ligand interactions (50). It is likely that different pathogens preactivate NK cells via distinct combinations of cytokines depending upon pathogen-specific TLR ligands stimulating particular myeloid cell populations (5, 22). Furthermore, heterologous infections may maintain cytokine preactivated NK cells; by contrast, MCMV-primed NK cells show very limited responses to bystander infections (49).

The impact of influenza vaccination on NK cell responses seems to reflect the kinetics of memory-like NK cell generation rather than availability of T cell help, because T cell IL-2 responses did not differ significantly over the course of the study. Cytokine-induced preactivation of NK cells for enhanced IFN-γ production could therefore be of significance in protection against influenza, with the extent of memory-like NK cell generation impacting on overall vaccine-induced protection. Further studies will be required to establish the relationship between memory-like NK cell generation by different vaccine formulations and subsequent responses to virus challenge.

## Supplementary Material

Data Supplement
