# Supplementary material for: Influenza Vaccination Generates Cytokine-Induced Memory-like NK Cells: Impact of Human Cytomegalovirus Infection
Source: J Immunol. 2016 May 27;197(1):313–25. doi: 10.4049/jimmunol.1502049 (PMC4911617; doi:10.4049/jimmunol.1502049)
Supplement: Data Supplement [file JI_1502049.zip › JI_1502049_Supplemental_Figures_1.pdf]

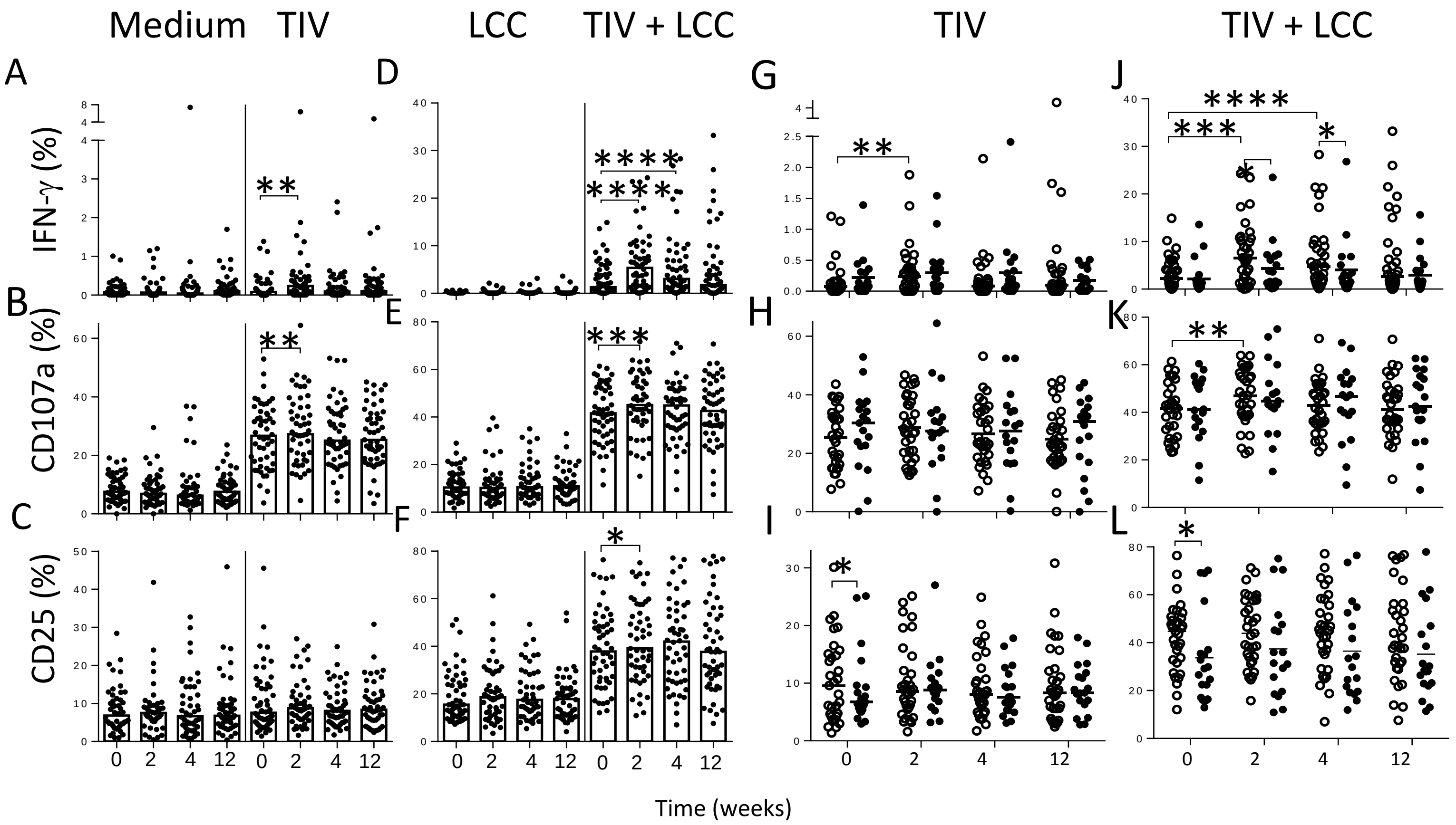

<u>Figure S1. NK cell responses to influenza viruses are enhanced after influenza vaccination, particularly among HCMV uninfected subjects.</u> PBMC were collected from 52 study subjects prior to (week 0) or after vaccination (weeks 2, 4 and 12) and cultured in vitro in culture medium alone or in the presence of Trivalent Influenza Vaccine (TIV, Split Virion, Sanofi-Pasteur) (A-C) alone or (D-F) in the presence of low concentrations of exogenous cytokines (LCC), IL-12 (12.5pg/ml) with IL-18 (10ng/ml). Expression of (A,D,G,J) IFN-γ, (B,E,H,K) CD107a and (C,F,I,L) CD25 were analysed within the total NK cell population. Responses were compared between from HCMV IgG seronegative (open symbols) (N = 33) and HCMV IgG seropositive individuals (closed symbols) (N = 19) after stimulation with (G-I) H1N1 virus alone or (J-L) in the presence of LCC. Statistical comparisons of NK cell responses before and after vaccination were performed using Wilcoxon signed rank test and between HCMV- and HCMV+ groups using Mann-Whitney U test, *p<0.05, **p<0.01, ***p<0.001.

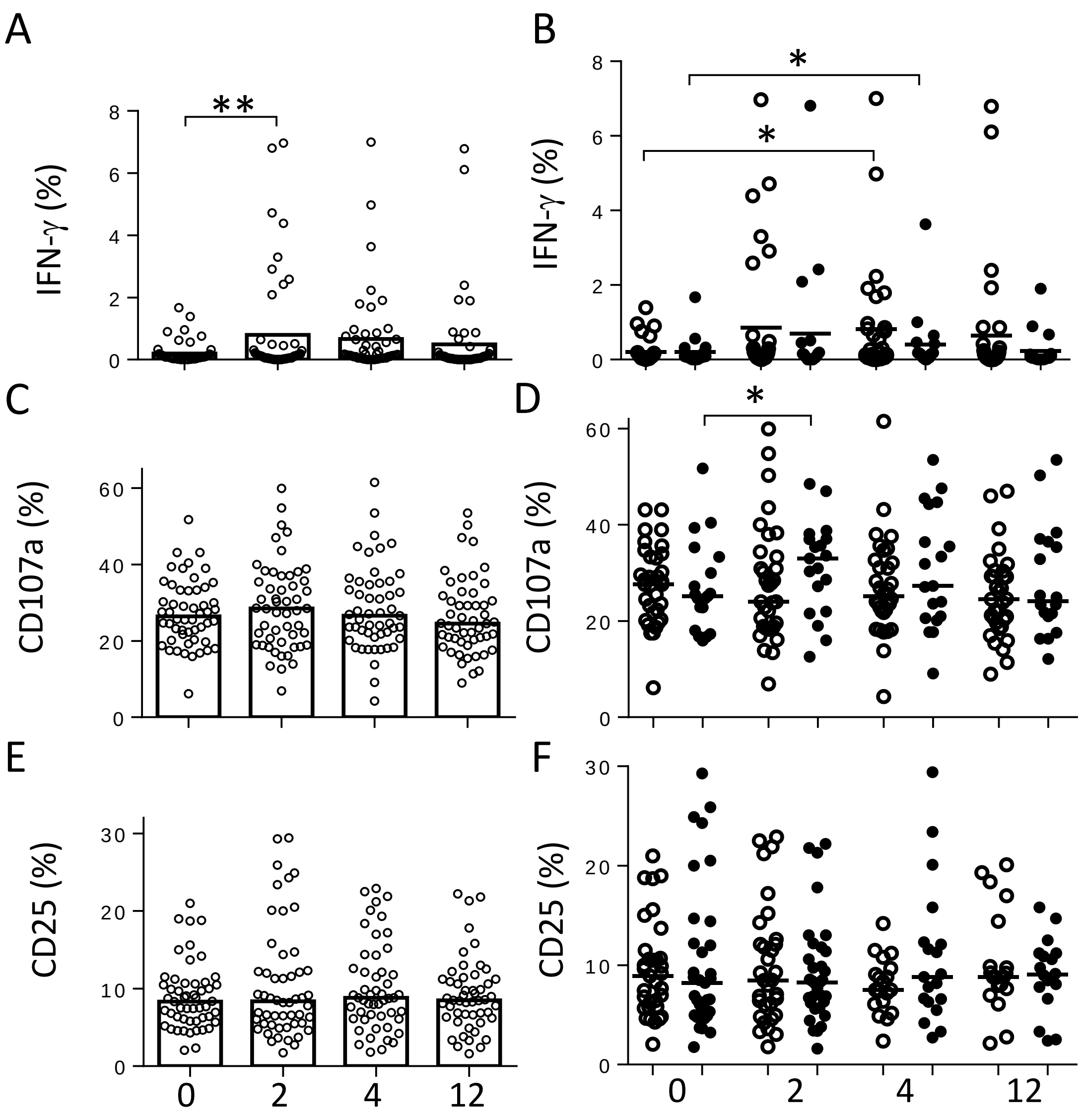

Figure S2. IFN-γ response to K562 cells after vaccination. NK cell expression of IFN-γ (A,D), CD107a (B,E) and CD25 (C,F) was determined in the entire cohort (A-C) and compared between HCMV seropositive (closed symbols symbols; N = 19) and seronegative (open symbols, N = 33) individuals (D-F). Paired statistical comparisons of NK cell responses before and after vaccination were performed using Wilcoxon signed rank test and inter-group comparisons using Mann-Whitney U test, *p<0.05, **p<0.01, ***p<0.001.

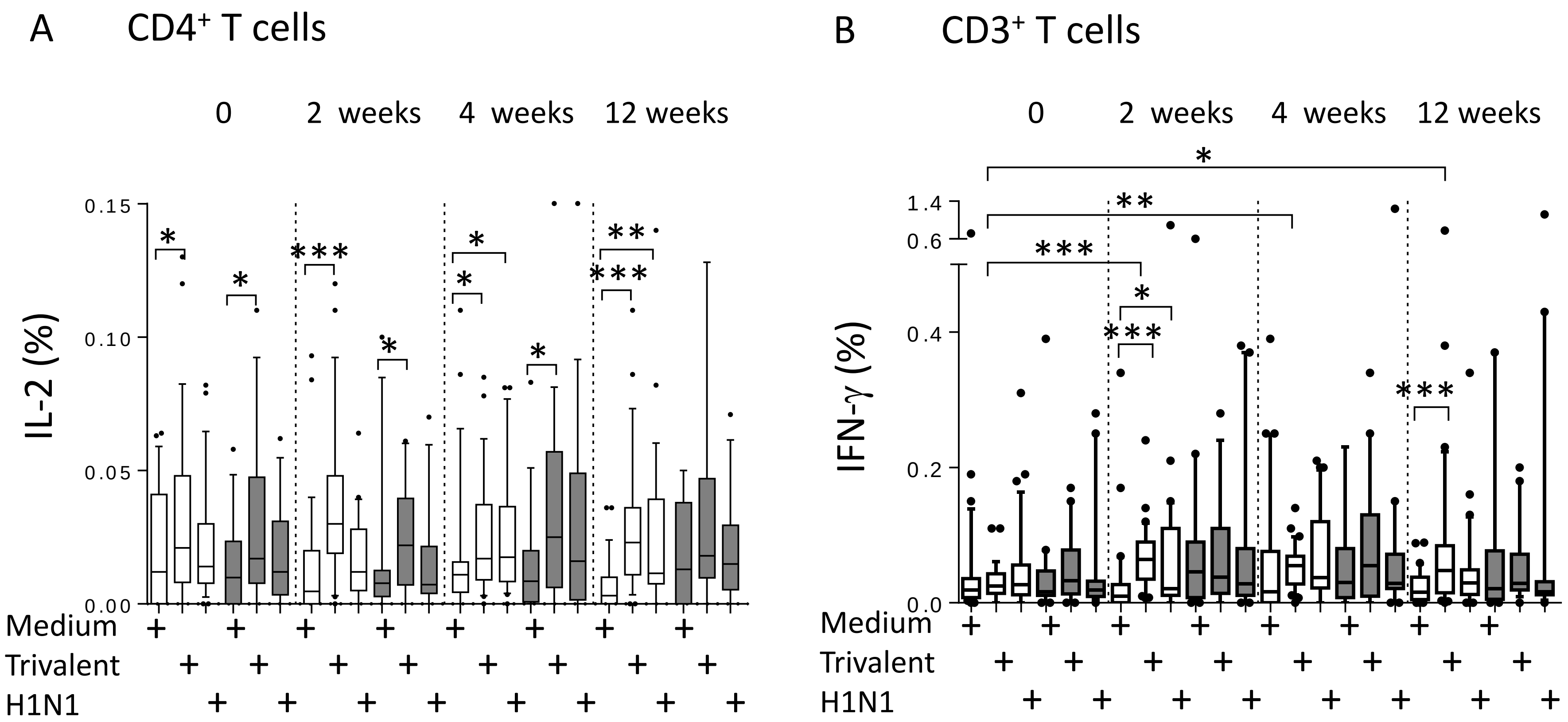

Figure S3. T cell IL-2 and IFN-γ responses to influenza viruses before and after vaccination.(A) Frequencies of $CD4^+$ T cells producing IL-2 and (B) $CD3^+$ T cells producing IFN-γ are shown for HCMV seronegative (open boxes; N = 33) and HCMV seropositive (closed boxes; N = 19) individuals across the vaccination time course. Boxes represent medians and interquartile ranges, whiskers represent 90th centiles and outliers are shown by solid dots. Paired comparisons were made using Wilcoxon signed rank test, *p<0.05, ** p<0.01, ***p<0.001.

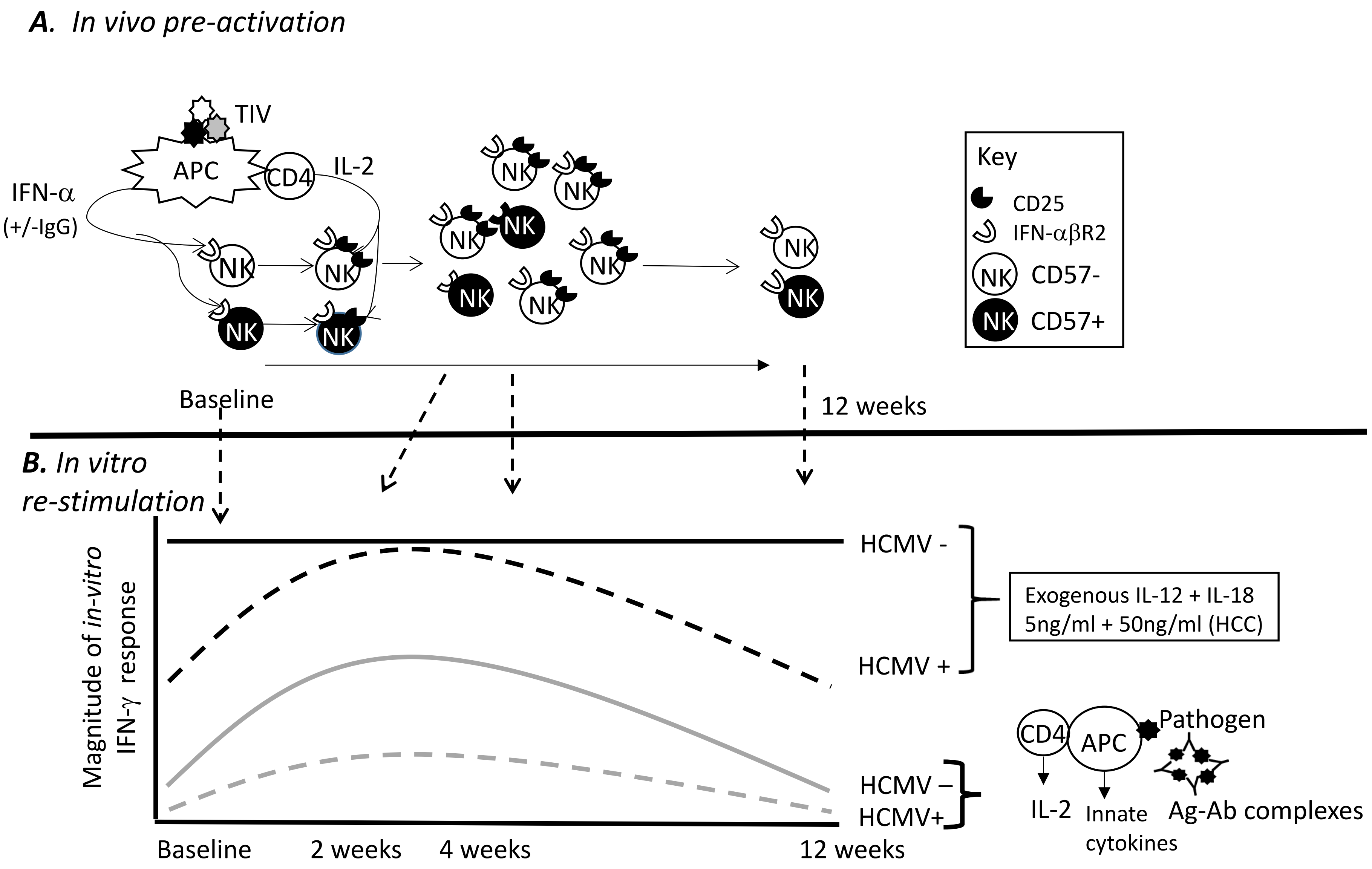

Figure S4. Model for generation of cytokine-induced memory-like NK cells after influenza vaccination.

A. We propose that vaccine-induced cytokines, including IFN-α, play a role in the generation of memory-like NK cells by mechanisms including upregulation of CD25 and other, as yet undefined, intrinsic changes. CD57- NK cells upregulate CD25 in response to vaccine-induced IFN-α and proliferate in response to IL-2 from influenza-specific CD4+T cells. After a period of weeks or months, in the absence of booster vaccination or infection, vaccine-induced memory-like NK cell populations lose CD25 and/or contract in number, returning to baseline levels.

B. In vitro re-stimulation reveals differences between HCMV- and HCMV+ individuals. The preponderance of CD57- NK cells in HCMV- individuals permits a robust IFN-γ response to high concentrations of exogenous IL-12 and IL-18 at all time points (solid black line). However, in HCMV+ individuals, vaccine induces the proliferation of CD57- NK cells resulting in enhanced IFN-γ responses to high concentrations of exogenous IL-12 and IL-18 (dashed black line). During re-exposure to the vaccine antigen/pathogen, innate cytokines, IL-2 and antigen-antibody immune complexes all contribute to NK cell activation (grey lines), but NK cells from HCMV- individuals (solid grey line) are more sensitive to these stimuli than are NK cells from HCMV+ individuals (dashed grey line).
